# ID 316 - Baixa Adesão das Revisões Sistemáticas Cochrane à Ferramenta RoB 2.0

**DOI:** 10.5327/2237-9622.2025.v34s1.316

**Published:** 2025-11-25

**Authors:** Lara Faria Souza Dias, Rafael Leite Pacheco, Ana Luiza Cabrera Martimbianco, Rachel Riera

**Keywords:** revisões sistemáticas, risco de viés, Cochrane, Cochrane *risk of bias table*

## Abstract

**Introdução:**

A versão 2 da ferramenta de risco de viés da Cochrane (RoB 2.0) é um dos instrumentos recomendados para avaliar o risco de viés em ensaios clínicos randomizados (ECR) incluídos em revisões sistemáticas Cochrane. A RoB 2.0, lançada em 2019, foi atualizada em 2011 e possui cinco domínios de viés. As principais diferenças entre a RoB 2.0 e a versão original (RoB) incluem: (i) aumento da complexidade dos itens avaliados, com o uso de questões sinalizadoras e mais opções de julgamentos; (ii) a necessidade de um algoritmo para sua aplicação; (iii) a exclusão de itens sobre viés de dados faltantes; e (iv) a incorporação de um julgamento geral de risco de viés, permitindo uma classificação única para o ECR. Apesar de ser uma ferramenta recomendada pelo Cochrane Handbook, estudos anteriores sugeriram uma baixa velocidade de implementação da ferramenta e, em 2022, apenas 24,1% das revisões Cochrane tinham adotado a RoB 2.0. Este estudo teve como objetivo determinar a adesão de todas as revisões Cochrane publicadas em 2023 à ferramenta RoB 2.0.

**Método:**

Metapesquisa conduzida pelo Núcleo de Ensino e Pesquisa em Saúde Baseada em Evidências e Avaliação de Tecnologias em Saúde (NEP-Sbeats), Universidade Federal de São Paulo (Unifesp). Foi realizada uma busca manual na Cochrane Library, com filtro de data, para identificar todas as revisões sobre intervenções em saúde publicadas nas edições de 2023.

**Resultados:**

Foram incluídas 380 revisões, das quais 18,16% (69/380) utilizaram a RoB 2.0 e 81,84% (311/380) adotaram a versão inicial da RoB Cochrane.

**Conclusão:**

A minoria das revisões Cochrane publicadas em 2023 aderiram à RoB 2.0. Apesar da melhoria na interpretação do viés e de sua influência nos resultados dos ECR, a RoB 2.0 tem uma estrutura mais complexa do que sua versão original, e há uma discussão crescente sobre sua aplicabilidade e usabilidade, o que parece limitar sua ampla adoção. Adicionalmente, também há discussões sobre a exclusão do domínio de avaliação de viés relacionado aos dados faltantes, o que poderia subestimar o viés de um desfecho ou do próprio ECR caso a RoB 2.0 não seja utilizada em conjunto com a ferramenta RoB-ME.

